# Role of Posttranslational Modifications of Proteins in Cardiovascular Disease

**DOI:** 10.1155/2022/3137329

**Published:** 2022-07-09

**Authors:** Yong-Ping Liu, Tie-Ning Zhang, Ri Wen, Chun-Feng Liu, Ni Yang

**Affiliations:** Department of Pediatrics, PICU, Shengjing Hospital of China Medical University, Shenyang, China

## Abstract

Cardiovascular disease (CVD) has become a leading cause of mortality and morbidity globally, making it an urgent concern. Although some studies have been performed on CVD, its molecular mechanism remains largely unknown for all types of CVD. However, recent in vivo and in vitro studies have successfully identified the important roles of posttranslational modifications (PTMs) in various diseases, including CVD. Protein modification, also known as PTMs, refers to the chemical modification of specific amino acid residues after protein biosynthesis, which is a key process that can influence the activity or expression level of proteins. Studies on PTMs have contributed directly to improving the therapeutic strategies for CVD. In this review, we examined recent progress on PTMs and highlighted their importance in both physiological and pathological conditions of the cardiovascular system. Overall, the findings of this review contribute to the understanding of PTMs and their potential roles in the treatment of CVD.

## 1. Introduction

Cardiovascular disease (CVD) is a common disease caused by multiple factors and is the disease with the highest mortality and morbidity in the world, and its incidence is still on the rise globally [1]. For example, between 2010 and 2020, deaths from CVD have increased by 12.5% accounting for 31% of all deaths worldwide [2, 3]. Owing to the increasing incidence of CVD, CVD-related deaths are expected to exceed 23.6 million globally by 2030 [4]. More than 95% of CVD-related deaths can be attributed to the following causes: hypertensive heart disease, stroke, ischemic heart disease (IHD), cardiomyopathy, atrial fibrillation (AF), and rheumatic heart disease (RHD) [5]. The high mortality and morbidity rates of CVD are serious threats to human health and affect the quality of life, necessitating research attention. Currently available treatment approaches have not been effective in treating CVD; moreover, the molecular mechanisms of CVD have not been fully elucidated. However, it is believed that posttranslational modifications (PTMs) of various proteins play important roles in the development of CVD.

Protein modification, also known as PTMs, refers to the chemical modification of specific amino acid residues after protein biosynthesis [6], which is an important biochemical process that occurs at the protein level [7]. PTMs mainly include acetylation, phosphorylation, succinylation, glutathionylation, and SUMOylation. It is estimated that approximately two-thirds of proteins in the body undergo PTMs. Notably, PTMs involves almost all aspects of cell biology by regulating signaling pathways to maintain cell homeostasis. Previous studies have confirmed that PTMs are considered to be a biological mechanism that regulates the function of proteome, affecting protein stability, activity, subcellular localization of proteins, and proteins' interactions [8]. Additionally, PTMs can regulate the catalysis and related modification sites of enzymes [9]. Recent studies have shown that PTMs are related to the pathogenesis of cardiovascular system [10]. Further study on the locus and effect of PTMs could be helpful to further elucidate the mechanism of CVD.

Therefore, this review aims to summarize the emerging roles of various PTMs in cardiovascular disease, discussing how PTMs affect the pathogenesis of CVD and provide future perspectives on CVD research.

## 2. The Common Types of PTMs in CVD

The common modification types of proteins in CVD mainly include acetylation, phosphorylation, succinylation, O-GlcNAcylation, methylation, ubiquitination, SUMOylation, glutathionylation, tyrosine nitration, and propionylation (Table 1). There may be a crosstalk among various PTMs in CVD. Notably, succinylation is also involved in the CVD, which is closely relative with metabolism in the cardiomyocyte [11]. Additionally, acetylation, which is another common type of PTMs, has been proved to participate in the regulation of enzyme activity [12]. The various types of PTMs and their modification sites have been described in detail in the previously published review, which we will not repeat here [13–15].

Various PTMs play important roles in CVD. Here, we take SUMOylation and phosphorylation as examples to illustrate the critical roles of PTMs in CVD. Phosphorylation affects the activity of protein, while SUMOylation may affect the production of protein quantity. Protein phosphorylation, a reversible PTMs process, is a PTM regulated by kinase and phosphatase and regulates protein activity and protein interaction through substrate phosphorylation and dephosphorylation [16]. Protein phosphorylation is a key regulator of nitric oxide synthase (eNOS) activity in endothelial cells. Phosphorylation at Tyr81 and Tyr657 regulates NO production by regulating the activity of eNOS. Notably, eNOS stabilizes the vascular environment with NO, and low doses of NO can maintain vascular tension. Therefore, phosphorylation of eNOS plays an important role in the dynamic regulation of eNOS activity in CVD [17]. Then, AKT is activated in cardiomyocytes by phosphorylation of T308 and S473 [18], mediates cellular metabolism, and plays a cardioprotective role in the heart. For example, Akt1 activity regulates cardiac systolic function and coronary angiogenesis and protects the heart from damage caused by pathological cardiac hypertrophy by maintaining physiological homeostasis [19]. In addition, oxidative phosphorylation (OXPHOS) is involved in the regulation of mitochondrial energy generation, and glutaredoxin-2 (Grx2) regulates the function of cardiac mitochondrial protein by catalyzing deglutathionylation [20, 21]. For instance, the study performed by Mailloux et al. found that Grx2-deficient mice had lower glutathionylation level, glucose intake, ROS production, and decreased mitochondrial complex I activity [21]. These changes can be reversed by glutathione (GSH) supplementation, restoring mitochondrial ATP production and reducing ROS production, which may be a novel treatment for redox damage.

In addition, SUMOylation is a reversible small ubiquitin-associated modification that binds lysine residues in proteins at the protein level. DeSUMOylation is performed by the SENP family [22]. Ubc9-mediated SUMOylation plays an important role in cardiac development, regulating the SUMO pathway and reducing the incidence of heart disease by increasing autophagy flux [23]. G protein signal transduction regulator 12 (RGS12) inactivates the AKT/mTOR signaling pathway by upregulating phosphorylation and SUMOylation of PTEN [24]. In endothelial cells, PPAR*γ* negatively regulates the eNOS-NO pathway through SUMOylation and exacerbates endothelial dysfunction [25]. Therefore, the imbalance of SUMOylation and deSUMOylation is associated with the development of CVD.

What is more, protein glutathionylation is a reversible oxidative modification in which a mixed disulfide is formed between glutathione and protein mercaptan to regulate protein activity. Glutathionylation is involved in the pathogenesis of CVD by regulating the redox signaling pathway, playing an increasingly important role in the regulation of cardiovascular function [26].

## 3. PTMs in Heart Development

**Figure 1:**
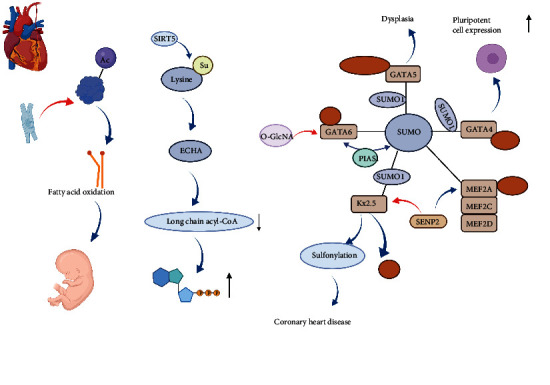
The role of posttranslational modifications in the process of heart development.

Congenital heart disease (CHD) is a genetically regulated congenital structural abnormality of the heart that is most common in neonates and before birth [27]. CHD, which accounts for about 1% of newborns, is more common in miscarriages and stillbirths [28]. Interactions between different types of proteins are the basis of normal heart development (Figure 1), and acetylation plays an important role in regulating embryonic heart development and myocardial cell energy metabolism [29]. The study conducted by Fukushima et al. reported that myocardial acetylation increases after birth, and cardiac hypertrophy reduces the acetylation level and delays the maturation of cardiac fatty acid oxidation [30]. In addition, succinylation is associated with neonatal cardiometabolism. Therefore, amino acid acetylation and succinylation have important effects on neonatal cardiac energy metabolism [31].

SUMOylation plays an important role in the development of a normal heart. Wang et al. reported that SUMO1 deficiency can cause cardiac septal dysplasia and congenital heart disease [32]. SUMOylation of GATA4, GATA5, and GATA6 regulates cardiac value and expression [33]. SUMOylation of GATA4 activates the modification of SUMO-1 at K445 and regulates the expression of cardiac protein in cardiogenic genes [34]. Furthermore, the SUMOylation of GATA5 in K324 and K360 restores abnormal expression of early cardiac differentiation and development [35]. The study conducted by Chen et al. found that the SUMO1 of GATA6 was translated into a SUMOylation at the K12 site, which regulated cardiac development through transcription [36]. These results suggest that the SUMOylation of GATA4, GATA5, and GATA6 may serve as therapeutic targets for early cardiac development. In addition, SUMOylation plays a key role in embryonic heart morphological development. SUMOylation promotes Nkx2.5 transcriptional activity [37]. SUMOylation enhances the activity of Nkx2.5 by covalency binding to the lysine residues 51, and Nkx2.5 mutations are involved in CHD through the abnormal SUMOylation [38]. Additionally, SENP2 interacts with Nkx2.5, and SENP2 overexpression promotes the severity of CHDs in the hearts of Nkx2.5 haploidy deficient mice [39]. Therefore, SUMOylation plays irreplaceable roles in the process of heart development.

In addition to SUMOylation, more and more recent studies have focused on glutathionylation, highlighting the critical role of SUMOylation in physiological conditions. Protein glutathionylation takes part in maintaining normal physiological functions of the cells and the body. For example, glutaredoxin-1 (Glrx) is an enzyme that can remove S-glutathionylation of proteins. Glrx reverses S-glutathionylation of GAPDH and plays a role in the key step of apoptosis signal transduction, thus participating in regulating apoptosis [40]. Moreover, eNOS is regulated by direct S-glutathionylation in the endothelial cells [41]. NO reacts with superoxide to form peroxynitrite, which activates S-glutathionylation in Cys674 to regulate SERCA2 activity and vascular smooth muscle function [42]. Nitroso glutathione (GSNO) reduces SIRT-1 activity by S-glutathionylation [43]. Consequently, S-glutathionylation plays a role in maintaining the physiological functions of endothelial cells and smooth muscle cells.

## 4. Role of PTMs in CVD

### 4.1. Role of PTMs in Myocardial Hypertrophy

**Figure 2:**
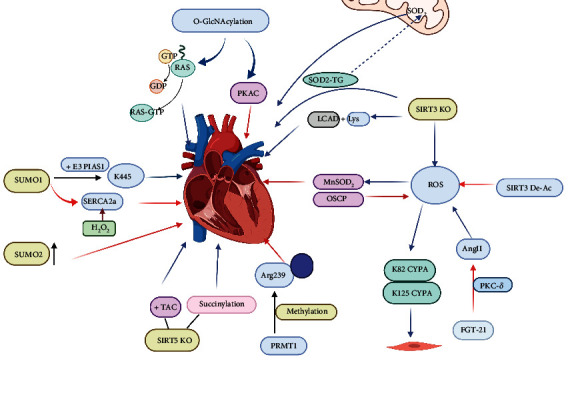
Specific modification sites of types of posttranslational modification in myocardial hypertrophy.

Cardiac hypertrophy is a process in which the heart maladjustment to various stimuli can progress to marked heart failure [44]. Acetylation is involved in the pathophysiology of cardiac hypertrophy (Figure 2). SIRT3 and SIRT1 acetylations influence the physiological and pathological states of the myocardium. Enhanced inflammation caused by ROS accumulation plays an important role in the progression of cardiovascular disease, and SIRT3 regulates mitochondrial function by regulating ROS metabolism and maintains normal cardiac function [45]. Similarly, the study carried out by Pillai et al. confirmed that ROS signal is a key signal of cardiac hypertrophy. Increased mitochondrial SIRT3 activity is associated with reduced acetylation of SIRT3 substrates, MnSOD, and oligomycin-sensitive endogenic protein (OSCP), which reduced ROS synthesis by increasing mitochondrial oxygen consumption rate, thereby preventing cardiac hypertrophy [46]. In addition, SIRT3 deficiency leads to acetylation of long-chain acyl-CoA dehydrogenase (LCAD) at lysine 42, resulting in disrupted fatty acid oxidation including reduced ATP production [47]. SIRT3 reduces intracardiac hypertrophy-related lipid accumulation and alleviates the progression of cardiac hypertrophy by deacetylation of LCAD [48]. In addition, SIRT1 is also involved in cardiac hypertrophy, and deacetylated protein kinase C-*δ* (PKC-*δ*) can improve cardiac hypertrophy [49]. Moreover, SIRT7 promotes the deacetylation of GATA4 to play an antihypertrophy role [50]. The study performed by Li et al. demonstrated that fibroblast growth factor 21 (FGF21) reduces angiotensin II-induced cardiac hypertrophy by increasing SIRT1 deacetylase activity [51].

Furthermore, O-GlcNAcylation has been implicated in the development of cardiac hypertrophy. Previous studies have shown that O-GlcNAcylation modification increases with the progression of cardiac hypertrophy, and O-GlcNAcylation promotes cardiac compensatory function during early cardiac hypertrophy [52]. Moreover, it has been observed that O-GlcNAcylation modulates vascular dysfunction due to intermittent hypoxia by regulating the MAPK pathway [53]. Therefore, O-GlcNAcylation plays a regulatory role in pathological hypertrophy.

Additionally, SUMOylation contributes to the regulation of oxidative stress response and improvement of cardiac function. SUMO-1 reduced oxidative stress markers in cardiac hypertrophy, and SERCA2a modified with SUMO-1 prevented the occurrence of cardiac hypertrophy [54]. Moreover, SUMO-1 activates the transcriptional activity of shock transcription factor 2 (HSF2) and induces cardiac hypertrophy in hypertensive rats through IGF-IIR-mediated signaling pathway [55]. SUMO-2 activates calcineurin- (Cn-) NFAT signaling and promotes cardiac hypertrophy [56]. Furthermore, the study performed by Wang et al. showed that the SUMOylation of C/EBP*β* K134 contributes to PARP1-induced cardiac hyperplasia by reducing the stability of C/EBP*β* [57].

Succinylation maintains cardiac metabolism and function under stressful conditions during cardiac hypertrophy. The study conducted by Hershberger et al. showed that SIRT5 participates in the deacetylation of protein substrates in cellular oxidative metabolism, maintaining cardiac oxidative metabolism under stress overload caused by cardiac hypertrophy to maintain mitochondrial energy production [58]. Similarly, the study carried out by Hershberger et al. demonstrated in SIRT5 KO mouse model that the succinylation level of oxidative metabolic protein increased and continued to accumulate after birth, inducing cardiac hypertrophy [59].

The S-glutathionylation participates in cardiac hypertrophy through oxidative stress. For example, the study performed by Pimentel et al. demonstrated that mechanical strain stimulates the Raf/MEK/ERK pathway, which was dependent on Ras s-glutathionylation, and, therefore, participates in myocyte growth signal transduction to regulate protein synthesis in cardiac hypertrophy [60]. In addition, glutathionylation was significantly increased in Grx2 knockout mice, leading to myocardial hypertrophy by reducing myocardial ATP production [21].

Methylation has also been implicated in the development of heart dysfunction. The study conducted by Jeong et al. suggested that PRMT1 is methylated at ATF4 of arginine 239 and regulates the protein stability of ATF4 in endoplasmic reticulum stress of cardiomyocytes and plays a protective role in cardiomyocytes [61]. In cardiomyocytes, PRMT5 symmetrically dimethylates HoxA9 and inhibits HoxA9 expression, preventing binding to promoters and protecting cardiomyocytes from cardiac hypertrophy [62]. PRMT5 protects cardiomyocytes from hypertrophy by reducing GATA4 transcriptional activity, and activation of PRMT5 may be a potential therapeutic target for cardiac hypertrophy [63]. Accumulation of methylated arginine metabolites can cause left ventricular diastolic dysfunction, which can aggravate myocardial hypertrophy injury. Therefore, arginine methylated derivatives, such as ADMA, can be used as markers to predict disease progression and prognosis [64]. However, more predictive targets for the disease need to be studied. Therefore, methylation is a potential target for treating future diseases.

In summary, acetylation, O-GlcNAcylation, SUMOylation, methylation, and glutathionylation are involved in the pathogenesis and progression of cardiac hypertrophy in response to oxidative stress, signaling pathways, and metabolites, thereby affecting normal cardiac function. However, the response levels of the new PTMs require further studies.

### 4.2. Role of PTMs in Acute Myocardial Infarction

Myocardial reperfusion injury is the injury during blood restoration after thrombolytic therapy after acute myocardial infarction, including myocardial tissue injury and dysfunction caused by reperfusion [65]. Epidemiology shows more than 800,000 people develop acute myocardial infarction each year, 27% of whom die, and myocardial infarction remains the leading cause of death globally [66].

Mitochondrial dysfunction is an important cause of myocardial reperfusion (I/R) injury. Clinical evidence suggests that patients with diabetes are more prone to cardiac I/R injury due to imbalance of antioxidant mechanism. The succinylation of SIRT5 is involved in the progression of I/R injury. The study performed by Boylston et al. illustrated that succinyl protein accumulates in cardiometabolic pathway and increases the severity of myocardial infarction through in vivo study of SIRT5 knockout mice after ischemia-reperfusion [11]. Moreover, it has been reported that SIRT5-deficient adipose-derived mesenchymal stem cells (ADMSCs) have increased protein succinylation, reduced mitochondrial respiration, and altered glucose metabolism patterns, thereby promoting vascular proliferation [67]. Succinylation of SIRT5 is a potential therapeutic target in I/R injury.

Phosphorylation is involved in the early activation of I/R damage. The study carried out Bibli and others reported that PYK2 is a key regulator of eNOS function in myocardial infarction. PYK2 activates phosphorylation of Y402 and eNOS activates phosphorylation of S1176. PYK2 leads to eNOS phosphorylation of Y656 induced by ischemia-reperfusion and regulates eNOS function in myocardial infarction [68]. Therefore, PYK2 may become a new therapeutic target for myocardial ischemia-reperfusion in the future.

Acetylation regulates I/R-impaired cardiac function. The study conducted by Leng et al. proposed that inhibition of HDAC6 modulates the acetylation of Prdx1 at K197, reducing ROS production and the severity of myocardial infarction and protecting against I/R injury [69]. Progressive loss of cardiomyocytes due to p53 is mediated apoptosis in myocardial infarction. In myocardial infarcted hearts, Lys118 residues of p53 are severely acetylated. However, it was completely reversed in the oxygenated heart, inhibiting Lys118 acetylation promoted the production of NOS3 and promoted the survival activity of p53, thus reducing myocardial infarction and protecting cardiac function [70]. Additionally, mangiferin increased FOXO3a deacetylation by upregulating SIRT1 in myocardial infarction, thereby preventing apoptosis and significantly reducing the size of myocardial infarction [71].

SUMOylation also implicates in AMI. PIAS1 is the specific E3 ligase of PPAR*γ* SUMOylation. After I/R injure, the levels of PIAS1 and PPAR*γ* SUMOylation decreased significantly, and apoptosis and inflammation increased, which exacerbated the severity of I/R injury [72]. Moreover, SENP2 deficiency increases Akt SUMOylation and Akt kinase activity, promoting cardiac regeneration [73]. Destruction of the SUMOylation of HDAC4 results in the accumulation of HDAC4 in cardiomyocytes, leading to a significant reduction in ROS [74]. Therefore, SUMOylation of HDAC4 could be a potential therapeutic target for I/R injury. Farnesoid X receptor (FXR) regulates cardiomyocyte apoptosis. Gao et al. demonstrated that reduced FXR SUMOylation promotes apoptosis and exacerbates cardiac injury by activating mitochondrial apoptosis pathway and autophagy dysfunction [75].

The role of glutathionylation in ischemic cardiomyopathy has also been reported. Glutathionylation of mouse cardiac SERCA protein leads to increased Ca^2+^ uptake and modulates myocardial contractility [76]. Actin glutathionylation promotes decreased systolic capacity during myocardial ischemia. Glutathionylation in mitochondrial complex I increases cytochrome C release, induces survival signals, promotes infarct size and cardiac dysfunction, and is regulated by glutathione deglutathione [77]. I/R injury leads to deglutathione in mitochondrial complex II and decreases its activity [78]. Therefore, glutathionylation of the proteins may be a potential therapeutic target for ischemic cardiomyopathy.

Overall, these findings indicated that succinylation, phosphorylation, SUMOylation, acetylation, and glutathionylation are involved in the formation and progression of I/R injury and play important roles in the regulation of cardiac repair.

### 4.3. Role of PTMs in Heart Failure

Heart failure (HF) is a pathological process in which cardiac systolic or diastolic dysfunction results in arterial hypoperfusion and impaired circulation [79]. According to statistics, there are about 37.7 million patients with heart failure worldwide, and the incidence of heart failure is still on the rise [80]. The high morbidity and mortality of heart failure have become a leading cause of hospitalization among adults and the elderly [80].

HF patients were divided into three types based on left ventricular ejection fraction (EF): reduced EF group (EF < 40%), borderline EF (40% ≤ EF < 50%), and the EF retention group (EF ≥ 50%). The decreased EF group suggested insufficient cardiac output, while the retained EF group suggested relatively normal left ventricular contractility [81].

GlcNAcylation plays an important role in heart failure. The O-GlcNAcylation levels depend on the activity of O-GlcNAc transferase and O-Glcnacase, and excessive O-GlcNAcylation can lead to cardiomyopathy and heart failure due to energy deficiency [82]. The reduction of O-GlcNAcylation may have a protective effect on overload induced pathological remodeling and heart failure. Midbrain natriuretic peptide prohormone (proBNP) levels are elevated, and Thr48 and 71 O-GlcNAcylation promote the secretion of proBNP in HF patients. Galna-transferase (GALNT) 1 and 2 promote the secretion of human proBNP by increasing glycosylation level, which can lead to heart failure [83]. Deglycosylated NT-proBNP level may be an important marker of heart failure [84].

Phosphorylation regulates energy metabolism in heart failure. Protein phosphorylation regulates SERCA2a activity and regulates myocardial diastolic and contractile processes. CAMP or cGMP dependent protein kinases are phosphorylated at Ser16, while Ca^2+^-calmodulin-dependent protein kinases (CaMKII) are phosphorylated at Thr17, which trigger SR Ca^2+^ uptake and SR Ca^2+^ load to regulate cardiac function in heart failure [85]. The study carried out by Copeland et al. confirmed the phosphorylation of MYBP-C plays an important role in regulating myocardial systolic function. In vitro studies have confirmed that MYBP-C can be phosphorylated at ser273, 282, 302, and 307. There are still several phosphorylation sites that need more in vivo studies to prove [86]. AMPK*α*2 enhances mitochondrial autophagy by phosphorylating PINK1 at Ser495, preventing further aggravation of heart failure [87]. Inhibition of PKC*ζ* reduces the formation of protein aggregates in cardiomyocytes; Bouvet et al. reported that phosphorylation activation of PKC*ζ* in heart failure leads to dysfunction of the proteolysis system, thereby exacerbating cardiac dysfunction [88].

Changes in acetylation status may play a role in the pathophysiology of heart failure. Gorski et al. demonstrated SIRT1 regulates cardiac SERCA2a activity through acetylation at site K492, and this site can activate SIRT1 deacetylation to restore SERCA2a activity through pharmacological action, thus playing an important role in cardiac homeostasis regulation. This site may be a new strategy for the treatment of heart failure [89]. SIRT1 alleviates HF by increasing the deacetylation level of p53 and inhibiting myocardial cell apoptosis [90]. In mitochondria, both fatty acid oxidation and complex I deficiency increase protein acetylation, exacerbating cardiac dysfunction and heart failure [91]. In the process of mitochondrial metabolic stress, SENP1 loss leads to the hypersumoylation of SIRT3 and the hyperacetylation of mitochondrial proteins, and the acetylation and SUMOylation crosstalk jointly regulate SIRT3 activation and mitochondrial metabolism [92]. Transforming growth factor *β*1 (TGF-*β*1) mediates the transformation of fibroblasts into myofibroblasts and increases extracellular matrix synthesis. SIRT3 deficiency leads to TGF-*β*1 expression and glycogen synthase kinase 3*β* (GSK3*β*) overacetylation at Lys15, leading to tissue fibrosis. SIRT3 deacetylates and activates GSK3*β*, thereby blocking TGF-*β*1 signal transduction and tissue fibrosis [93]. Therefore, SIRT3 is a key regulator of cardiac energy metabolism and a potential therapeutic target. The study performed by Renguet et al. showed that the acetylation of leucine inhibits cardiac glucose uptake and dysregulated glucose homeostasis [94]. Further studies are needed to regulate the acetylation of mitochondrial proteins by more metabolites related to oxidative metabolic pathways.

The SUMOylation of SERCA2a also plays an important role in HF. SERCA2a levels decrease in HF and regulate intracellular calcium homeostasis, which has potential clinical application value [95]. Therefore, SERCA2a is SUMOylation at Lys 480 and 585 and regulates myocardial contractility in heart failure [96]. Additionally, the SUMOylation of SERCA2a has also been reported in the treatment of HF, for example, simultaneous transfection of SUMO1 and SERCA2a with adeno-associated virus vectors also increased SERCA2a expression and function [97]. Therefore, SENP5 is the only SENP upregulated in human heart failure. The SUMOylation of Drp1 leads to increased mitochondrial swelling and fission, triggering apoptosis, leading to heart failure and cardiomyopathy [98].

Overall, these findings indicated that phosphorylation, acetylation, O-GlcNAcylation, and SUMOylation are involved in the pathogenesis of HF and the regulation of heart remodeling. However, further studies are necessary to elucidate the crosstalk between PTMs in treating HF.

### 4.4. Role of PTMs in Hypertension

Hypertension is a multifactorial disease involving the heart, kidney, and other systems [99], whose vascular damage causes serious damage to the heart and is the main risk factor for cardiovascular diseases. Hypertension was the fourth leading cause of cardiovascular diseases in the world in 2015, accounting for one-third of global deaths [100]. Vascular dysfunction is an important pathophysiological mechanism of hypertension and endothelial cell dysfunction throughout the development of hypertension [101].

**Figure 3:**
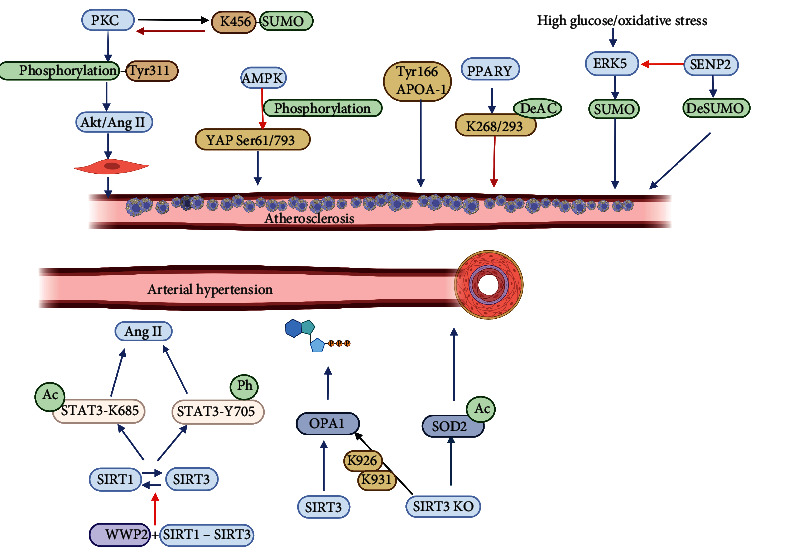
The role of posttranslational modifications in atherosclerosis and hypertension.

Phosphorylation is involved in the development of hypertension (Figure 3). WWP2 regulates hypertensive vascular disease by regulating SIRT1-STAT3 complex. In vascular smooth muscle cells, WWP2 and SIRT1-STAT3 complexes promote the acetylation of STAT3-K685 and phosphorylation of STAT3-Y705 by inhibiting the interaction between SIRT1 and STAT3 and increase the severity of hypertensive lesions [102]. The study carried out by Boguslavskyi et al. illustrated that phospholemman is hypophosphorylated in a mouse model of aging that induces senescent essential hypertension. Phosphorylation of phospholemman therefore regulates vascular tone and hypertension [103]. Antihypertensive drugs can inhibit the phosphorylation of epidermal growth factor receptor (EGFR) of Y1068 in vascular smooth muscle cells. Therefore, EGFR inhibition is a treatment for cardiovascular diseases such as hypertension and atherosclerosis [104].

Acetylation-mediated mitochondrial metabolism is involved in the development of hypertension. The normal morphology of mitochondria is regulated by the balance of fusion and fission. The study performed by Samant et al. reported that the acetylation of the mitochondrial fusion protein OPA1 at lysine 926 and 931 occurs. Excessive acetylation of OPA1 under pathological stress reduces the GTPase activity of OPA1. Mitochondrial deacetylase SIRT3 can deacetylate OPA1, improve its GTPase activity, and restore mitochondrial function [105]. Loss of SIRT3 promotes endothelial dysfunction, oxidative stress, and vascular hypertrophy in essential hypertension, leading to cardiac dysfunction [106]. Cyclophilic protein A (CyPA) increases VSMC and endothelial cell production by acetylation of lysine K82 and K125 [107]. Similarly, SIRT3 loss leads to high acetylation of mitochondrial SOD2, leading to endothelial dysfunction, hypertension, and other cardiovascular diseases in mice [106].

Moreover, the level of O-GlcNAcylation affects vascular function. O-GlcNAcylation regulates vascular reactivity under normal physiological conditions. Dysvascular dilation is associated with elevated levels of O-GlcNAcylation protein in hypertensive patients [108]. However, the acute increase of the O-GlcNAcylation of the protein inhibits the expression of iNOS and prevents vascular dysfunction induced by TNF-*α*, thereby protecting the heart and blood vessels [109]. In blood vessels, eNOS and PKC are targets of O-GlcNAcylation in the vascular system [110]. The study carried out by Nagy et al. demonstrated that increased O-GlcNAcylation inhibited angiotensin II-induced Ca2^+^ levels in ventricular myocytes [111]. Endothelin-1 increases O-GlcNAcylation levels and plays a role in vascular changes in hypertension-related vascular dysfunction [112].

Furthermore, SUMOylation has been also been implicated in hypertension. SENP3 inhibits proteasome-dependent degradation through the deSUMOylation of *β*-catenin. Mediate vascular remodeling and regulate vascular homeostasis [113]. Activating transcription factor 3 (ATF3) is SUMOylation of lysine 42 via SUMO1 and is involved in Ang II-induced endothelial dysfunction [114]. Therefore, SUMO2 plays an important role in maintaining normal endothelium-dependent vascular function [115]. The study carried out by Kim et al. reported that overexpression of SUMO2 would increase oxidative stress, damage vascular endothelial function, and increase the incidence of hypertension [116]. SUMO2 enhancement can lead to apoptosis, disrupt vascular physiological state, and lead to endothelial dysfunction. Therefore, an imbalance in SUMOylation could lead to a pathological state.

In hypertensive vessels, increased eNOS S-glutathionylation in endothelial cells is positively associated with impaired endothelium-dependent vasodilation, which is reversible and can be reversed with thiol-specific reducing agents [117]. S-glutathionylation of small GTPase Ras plays a role through Ser mutants that increase the muscle tone of AngII-induced smooth muscle cells and regulate hypertension [118]. Thus, S-glutathionylation regulates the progression of hypertension by acting on endothelial and smooth muscle cells.

Overall, these findings confirmed that acetylation, phosphorylation, O-GlcNAcylation, SUMOylation, and S-glutathionylation are involved in the pathogenesis of hypertension.

### 4.5. Role of PTMs in Atherosclerosis

Atherosclerosis is characterized by the formation of lipid plaques in the arteries [119], which leads to hardening of the wall, narrowing of the lumen, and reduced elasticity, leading to ischemic cardiovascular disease. Atherosclerosis leads to myocardial infarction, stroke, and other peripheral artery-related diseases with high morbidity and mortality [120] and has become the leading cause of mortality worldwide [121].

Acetylation has been implicated in atherosclerosis development. In a case-control study of 342 patients, An et al. reported that glycoprotein acetylation level was positively correlated with the incidence of heart disease [122]. Thiazolidinedione (TZDs) is a peroxisome proliferator activated receptor *γ* (PPAR*γ*) agonist, which is an antiatherosclerosis insulin sensitizer. The study conducted by Liu et al. illustrated that PPAR*γ* was deacetylated at K268 and K293 and inhibited the expression of atherosclerotic factors through endothelium-dependent vasodilation, thus exerting an antiatherosclerosis effect [123]. Aspirin acts as an antiplatelet agent by preventing protein aggregation through acetylation [124]. Hyperglycemia has a direct effect on the acetylation of COX-1 at Ser529, and aspirin can inhibit its acetylation and play a protective role in diabetes-induced cardiovascular disease [125].

Tyrosine nitration of proteins increases the likelihood of atherosclerosis. The study performed by Peluffo and Radi suggested that nitrotyrosine was a new independent marker of cardiovascular disease [126]. The study carried out by DiDonato et al. illustrated that nitration of ApoA-I at Tyr166 in atherosclerotic arterial wall leads to vascular selective dysfunction [127]. Furthermore, nitrification of PKG-1*α* at tyrosine 247 and 425 and nitrification of tyrosine 247 can reduce the affinity of PKG-1*α* to cGMP and regulate the proliferation and differentiation of atherosclerosis smooth muscle cells [128].

Phosphorylation is essential for atherosclerosis. Endothelial barrier dysfunction is one of the causes of atherosclerosis. The study conducted by Starosta et al. reported that high OXPAPC concentrations promote Src and ROS-dependent phosphorylation of VE-cadherin Tyr658 and Tyr731, leading to endothelial barrier dysfunction [129]. AMPK phosphorylates YAP at sites 94, 61, and 793 on serine, where Ser61 and Ser793 inhibit YAP activity, regulate cell metabolism and function, and participate in anticardiovascular disease effects [130]. PKC activation is required for Ang II-induced migration of vascular smooth muscle cells (VSMCs). PKC is rapidly phosphorylated by Ang II at Tyr311 and contributes to the development of Ang II-induced cardiovascular disease by participating in Akt activation and Ang II-induced VSMC hypertrophy [131]. The study performed by Wang et al. showed that coal-derived PM2.5 can promote the phosphorylation of MAPK signaling pathway-related proteins and promote the formation of atherosclerosis [132].

Atherosclerosis is associated with S-glutathionylation. The previous study showed that serum S-glutathionylation levels were positively correlated with the severity of atherosclerotic disease and could be used as a predictive marker of arteriosclerotic obliterans [133]. Cell oxidized low-density lipoprotein (OxLDL) is a major risk factor for atherosclerosis. OxLDL promotes glutathione formation of related proteins glutathionylation and ROS and promotes cell death [134]. Angiotensin II regulates redox reactions by forming and activating S-glutathionylation of Ras via Cys118, thereby inducing protein synthesis in atherosclerosis [118]. In addition, p21Ras is modified by S-glutathionylation at Cys118 to regulate growth factor signaling in the vascular system. S-glutathionylation of IP3 receptor regulates Ca2^+^ storage and Ca2^+^ release and influences the Ca2^+^ homeostasis of endothelial cells [135]. However, the mechanism of S-glutathionylation in atherosclerosis has not been fully elucidated and needs further study.

Furthermore, SUMOylation is involved in endothelial dysfunction, dyslipidemia, and VSMC proliferation in atherosclerosis. SUMOylation regulates the release and expression of inflammatory factors by modifying NF-*κ*B and TLR. GATA2 was also modified to regulate the expression of endothelial cell adhesion molecules and jointly regulate the inflammatory progression of atherosclerosis [136]. ERK5 is an anti-AS cytokine, and SUMOylation of ERK5 contributes to endothelial dysfunction by inhibiting the expression of nitric oxide synthase in endothelial cells, leading to atherosclerosis [137]. However, overexpression of p53 and ERK5 SUMOylation in endothelial cells protects atherosclerosis by inhibiting apoptosis and adhesion molecules. SENP2 regulates p53 and ERK5 SUMOylation and is involved in endothelial atherosclerosis [138]. SUMOylation of NLRP3 inhibits the activation of inflammasome. SENP7 deficiency inhibits the SUMOylation of NLRP3, which activates caspase-1 and releases IL-1*β*, and exacerbates the inflammatory damage of atherosclerosis [139]. In addition, the SUMOylation of PAR*α* at K185 is closely associated with atherosclerotic dyslipidemia [140]. Therefore, SUMO and SUMOylation play a crucial role in atherosclerosis, and SUMOylation of ERK5, SENP2, and GATA2 may be potential therapeutic targets for atherosclerosis.

Overall, the current studies have proved that acetylation, phosphorylation, tyrosine nitration, SUMOylation, and S-glutathionylation are involved in the development of atherosclerosis, providing potential targets for future clinical research.

## 5. Future Development Direction

Recent advances in mass spectrometry and omics techniques have facilitated studies on PTMs and crosstalk between PTMs of proteins. PTMs not only affect the progression of CVDs but also regulate CVD-related syndromes, such as insulin sensitivity and diabetic cardiomyopathy. For example, the study carried out by Choi et al. demonstrated that PTMs of PPAR*γ* regulate PPAR*γ* activity or stability, thereby optimizing PPAR*γ* activity and reducing insulin resistance [141], which could improve the treatment of metabolic syndromes and atherosclerotic vascular diseases. Moreover, SUMOylation is involved in some kinase pathways that regulate apoptosis in diabetic cardiomyopathy [142]. The study conducted by Heo et al. demonstrated that SUMOylation regulates ERK5 by inhibiting ERK5's transcriptional capacity [138], thereby regulating apoptosis. Furthermore, Tabrez et al. used an untargeted metabonomics approach to identify metabolites that may be associated with CVD [143], indicating the importance of metabonomics in CVD treatment. However, further research is needed to determine the relationship between metabolites and the pathogenesis of CVD. The study performed by Sadhukhan et al. illustrated that succinylation and SIRT5 are important regulators of cardiac function, using metabolomics with proteomics techniques [144].

Mass spectrometry provides a method to further investigate the pathogenesis of metabolite-associated PTMs in cardiovascular diseases. Additionally, sequencing technology and the cosequencing of RNA and protein levels have improved the understanding of the role of PTMs in CVDs. For example, machine learning-based prediction of protein modification sites, such as acetylation, succinylation, and ubiquitination, has emerged as predictive tools of CVDs [145]. Although studies on PTMs have increased recently, the role of PTMs in the pathogenesis of atherosclerosis in CVD requires further studies.

Furthermore, combination of proteomics and precision medicine can promote the identification of candidate biomarkers for clinical diagnosis, prognosis, monitoring, and personalized patient medication design and delivery, which can promote CVD treatment. For example, the study performed by Meng et al. illustrated the use of the target space of PTM protein isomers to inspire new directions in drug design by precisely targeting PTM protein isomers or complexes that are highly related to biological functions [146]. This new approach will further promote personalized therapy for precise treatment of PTM subtypes.

Several studies have identified the role of PTMs of protein in CVDs, and specific-related modification sites may become new biomarkers for predicting cardiovascular disease risk. The study carried out by Kuwahara et al. identified that deglycosylated NTproBNP as a significant new marker of heart failure [84]. Additionally, kinase-related drug development, such as the mitochondrial membrane protein branch *α*-ketoate dehydrogenase (BCKD) complex, which regulates mitochondrial oxidative metabolism through BCKD kinase modification sites, could be a potential therapeutic target for CVD. HDAC inhibitors can increase lysine acetylation, and HDAC inhibition therapy could be potential treatment strategy for patients with heart failure [147]. PTMs are based on basic research on clinical guidance methods to provide a theoretical basis for clinical diagnosis, prognosis, and treatment of CVD.

Currently, the development and clinical trials of therapeutic drugs targeting PTMs mainly focus on phosphorylation. For example, alamandine is a peptide of the renin-angiotensin system (RAS) that protects the cardiovascular system and improves stress-overload induced cardiac remodeling by reducing ERK1/2 phosphorylation and increasing AMPK*α* phosphorylation [148]. Alamandine holds promise as a cardiovascular treatment in the future. In addition, immunosuppressant applications are being developed to enhance the monitoring of therapeutic agents and individual differences through pharmacodynamics (PD) monitoring by directly measuring the activity of target enzymes and phosphorylation. The study conducted by Budde et al. determined the activity of mycophenolate acid target enzyme inosin-5′-monophosphate dehydrogenase (IMPDH) and the phosphorylation of rapamycin (mTOR) target molecule and found that the mTOR pathway is an appropriate target for individualized PD monitoring of sirolimus and everolimus after organ transplantation [149]. Moreover, studies on mitochondrial drug targets in CVD mainly focus on mitochondrial ATP-dependent potassium channel. Diazoxide activates mitochondrial ATP-dependent potassium channel phosphorylation by stimulating PKC, promotes channel opening, and plays a protective role in myocardial ischemia-reperfusion injury [150]. However, the multicenter clinical studies are needed to further prove the clinical effectiveness. More potential therapeutic targets require pharmacodynamic detection and clinical trials to prove their potential clinical therapeutic value.

## 6. Conclusion

PTMs is not only involved in protein stability, activity, subcellular localization, and interaction but also plays an important role in regulating protein expression under physiological and pathological conditions. PTMs play an important and complex role in regulating the progression of CVD. The protein may have multiple modification sites, and there is crosstalk among various PTMs. PTM sites may be potential drug targets, and the development and utilization of these signaling pathways may become new therapeutic strategies. Therefore, an in-depth understanding of PTMs and its mechanism of crosstalk in the pathological process can provide basic theoretical basis for clinical application. Further research is needed to improve the understanding of CVD in young adults and treatment strategies.

Therefore, future work on the involvement PTMs in CVD might include the following:

1. The specific mechanism of SIRT5 as a regulatory factor of CVD in heart development and the role of succinylation in heart development
2. Further studies on the cytodynamics of O-GlcNAC modification, and the signaling pathway and mechanism of regulating specific proteins in vascular vessels
3. The potential clinical application of acetylation in diabetic cardiomyopathy was elucidated by sequencing and metabolite analysis
4. Further studies on the mechanism and physiological consequences of crosstalk among various PTMs
5. Clinical studies on the potential therapeutic targets of PTMs in CVD, and precision medicine for clinical diagnosis, prognosis, and treatment of CVD
6. Further study on the action sites and related mechanisms of various novel modifications in CVD, including crobitoylation, 3-hydroxybutyrylation, and malonylation

## Figures and Tables

**Table 1:** Summary of known modification types and loci in cardiovascular disease.

Modification	Chemical structure	Modified residue	Addition	Removal	Type of CVD
Acetylation	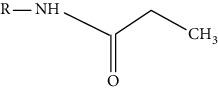	Lysine, N-termini	Enzymatic and nonenzymatic	Enzymatic (HDACs and SIRT1-7)	Myocardial hypertrophy, HF, I/R, hypertension, atherosclerosis
Phosphorylation	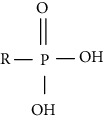	Serine, threonine, tyrosine	Enzymatic (kinase, phosphatase)	Enzymatic (kinase, phosphotransferase)	HF, I/R, hypertension, atherosclerosis
Succinylation	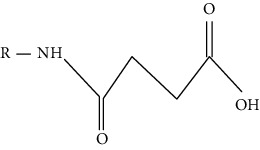	Lysine	Enzymatic and nonenzymatic	Enzymatic (SIRT5)	I/R, AF, mycordial hypertrophy
O-GlcNAcylation	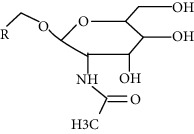	Serine, threonine cysteine	Enzymatic (OGT)	Enzymatic (OGA)	HF, hypertension, myocardial hypertrophy
Methylation	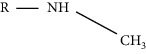	Lysine, N-termini	Enzymatic (methyltransferases)	Enzymatic (demethylases and KDMs)	HF, myocardial hypertrophy
Ubiquitination	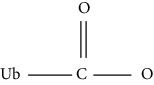	Lysine	Enzymatic (E1/E2/E3)	Enzymatic (deubiquitinating enzymes)	HF
SUMOylation	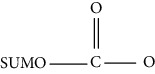	Lysine	Enzymatic (E1/E2/E3)	Enzymatic (deSUMOylation enzyme)	Myocardial hypertrophy, HF, I/R, MI, hypertension, atherosclerosis, cardiac remodeling
Glutathionylation	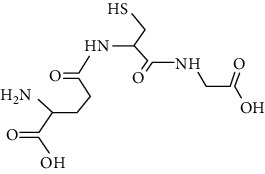	Cysteine	Glutenoxyredox protein	Glutaredoxin-1	Myocardial hypertrophy, I/R, MI, hypertension, atherosclerosis, cardiac remodeling, HF
Tyrosine nitration	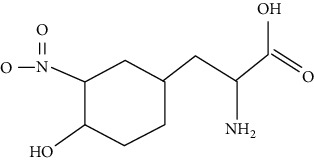	Tyrosine	Structure change	Nonenzymatic	I/R, HF, atherosclerosis
Propionylation	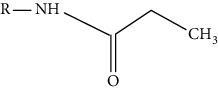	Lysine	Enzymatic	Enzymatic (SIRT1-3)	MI, atherosclerosis
